# Quantification of Ground Glass Opacities Can Be Useful to Describe Disease Activity in Systemic Sclerosis

**DOI:** 10.3390/diagnostics10040225

**Published:** 2020-04-16

**Authors:** Domenico Sambataro, Gianluca Sambataro, Francesca Pignataro, Wanda Maglione, Lorenzo Malatino, Carlo Vancheri, Michele Colaci, Nicoletta Del Papa

**Affiliations:** 1 Artroreuma S.R.L., Outpatient of Rheumatology Associated with the National Health System corso S. Vito 53, 95030 Mascalucia, Italy; d.sambataro@hotmail.it; 2 Department of Clinical and Experimental Medicine, Internal Medicine Unit, Cannizzaro Hospital, University of Catania, via Messina 829, 95100 Catania, Italy; malatino@unict.it (L.M.); michele.colaci@unict.it (M.C.); 3 Regional Referral Centre for Rare Lung Diseases, A. O. U. “Policlinico-Vittorio Emanuele” Department of Clinical and Experimental Medicine, University of Catania, 95123 Catania, Italy; vancheri@unict.it (C.V.); nicoletta.delpapa@asst-pini-cto.it (N.D.P.); 4 Scleroderma Clinic, Department of Rheumatology, ASST G. Pini-CTO, 20122 Milan, Italy; francy.pignataro@hotmail.it (F.P.); maglionewanda@gmail.com (W.M.)

**Keywords:** systemic sclerosis, interstitial lung disease, lung fibrosis, ground glass opacities, honeycombing, high resolution computed tomography, disease activity, NEMO score, Wells score, disease severity

## Abstract

Interstitial lung disease (ILD) is the main cause of death in systemic sclerosis (SSc) patients. Usually, patients have lung involvement characterized by ground glass opacities (GGOs), but honeycombing (HC) is also possible. The Wells score is a semi-quantitative index, which is able to assess ILD by distinguishing its main components. The aim of this work is to evaluate the Wells score in relation to the disease activity (DA) index. We enrolled 40 consecutive SSc-ILD patients (26 diffuse cutaneous form, dcSSc, and 14 limited form, lcSSc). All patients were evaluated by the European Scleroderma Study Group (ESSG) index, high-resolution computed tomography, transthoracic echocardiogram, pulmonary function tests (PTSs), and nailfold videocapillaroscopy for the number of microhemorrhages (NEMO) score. In our study, the total extent of ILD (TE-ILD), fibrosis and GGOs correlated with dyspnea (*p* = 0.03, 0.01 and 0.01 respectively), but not with the ESSG index. Considering only the dcSSc patients, TE-ILD and GGOs correlated with the ESSG index (r = 0.5 *p* = 0.009), while fibrosis grade correlated with disease duration and systolic pulmonary artery pressure. In conclusion, our data suggest that GGO correlates with DA, while fibrosis may be a sign of disease damage. The quantification of pulmonary involvement using the Wells score can be a useful tool for assessing the appropriate treatment in SSc patients.

## 1. Introduction

Systemic sclerosis (SSc) is a connective tissue disease characterized by vasculopathy, Raynaud’s phenomenon (RP) and fibrosis that involves skin and internal organs.

Pulmonary involvement is the primary cause of death in SSc patients [1]. Interstitial lung disease (ILD) occurs in over 90% of patients and 40% of them show restrictive changes in pulmonary function tests [2]. The use of high resolution computed tomography (HRCT) instead of conventional radiography has significantly improved sensitivity in the evaluation of pulmonary involvement [3], providing additional and more definite information about lung damage. The most common HRCT pattern observed in SSc is nonspecific interstitial pneumonia (NSIP), with a lower degree of coarse reticulations [4] and a greater proportion of ground-glass opacities (GGOs), which can reflect a potentially reversible inflammatory infiltration [5]. In addition, it is not that uncommon in SSc patients to observe GGOs together with honeycombing (HC), the main feature of usual interstitial pneumonia (UIP). HC may be prevalent in about 10–20% of SSc patients, depicting a UIP pattern. Despite the known difficulties in finding an ILD-reverting treatment, GGOs might represent a clinical and therapeutic challenge, in comparison with the irreversible HC features considered as the structural damage progression from GGO lesions [6,7]. This means that HRCT could be considered as not only a very useful tool for the diagnosis of pulmonary involvement, but also for the prognosis. Indeed, Goh proposed a very easy quantitative evaluation of ILD, discriminating a clearly <20% pulmonary involvement from a clearly >20% one [8]. This cut-off is predictive of mortality in systemic sclerosis.

In this view, the quantification of the disease, and above all, of its potentially reversible feature (GGOs), can be very useful in the clinical management of these patients. Several indexes have been studied for ILD-SSc, however, computer-aided quantification has difficulty in distinguishing the two main components of ILD. On the contrary, the Wells score, despite its semi-quantitative design, is able to produce a quantification of the total extent of ILD (TE-ILD) and a proportion of GGOs and fibrosis (HC) in ILD-SSc [9].

Therefore, the purpose of this study is to quantify pulmonary involvement by means of this score in a cohort of systemic sclerosis patients, looking for any correlation with disease activity (DA) and clinical features.

## 2. Materials and Methods

We enrolled 40 (4 males, 36 females) consecutive patients, with a diagnosis of SSc according to the American College of Rheumatology/European League Against Rheumatism (ACR/EULAR) classification criteria [10], who presented a clinical indication for pulmonary imaging. These patients were also classified as having a diffused (dcSSc) or limited (lcSSc) form of the disease according to their skin involvement [11], and as having an early or longstanding disease, on the basis of disease duration. Disease stages were defined as suggested by Medsger and Steen: for early lcSSc, disease duration was <5 years; for intermediate/late lcSSc, disease duration was ≥5 years; for early dcSSc, disease duration was <3 years; and for intermediate/late dcSSc, disease duration was ≥3 years [12].

Clinical assessment was performed according to the European Scleroderma Study Group (ESSG) criteria [13,14], which we used as the gold standard for DA in SSc. This score takes into account the following parameters to define DA: modified Rodnan skin score (mRSS) >20, presence of scleredema, digital necrosis, hypocomplementaemia (C3 and/or C4), worsening of skin, articular/muscular function and cardiopulmonary symptoms, erythrocyte sedimentation rate (ESR) >30 mm/h, and diffusion lung for carbon monoxide (DLCO) <80% of the predicted value. During the clinical assessment, mRSS was evaluated by at least two rheumatologists experienced in the management of SSc, and the final value was intended as a mean of the two evaluations. Good concordance was reported in the evaluation of mRSS by two different, blinded rheumatologists.

For the radiological assessment, all patients underwent a HRCT with a thickness ranging between 0.625 and 1.25 mm. The presence of GGO and HC, as well as the description of prevalent patterns, were performed by experienced pulmonologists and radiologists according to the current guidelines [15]. The quantification of pulmonary disease was made according to the Wells score [9]: the scans were reviewed in five levels corresponding to the origin of the great vessels, carina, pulmonary venous confluence, one centimetre above the right hemidiaphragm and between the lasts two levels. In each level, a quantification was made of the overall extent of ILD (both reticular pattern and GGOs), the percentage of GGOs in relation to ILD, the coarseness of fibrosis in a semiquantitative manner (0 = only GGOs, 1 = fine intralobular fibrosis, 2 = microcystic reticular patterns compromising air spaces of no more than 4 millimeters, 3 = macrocystic reticular patterns compromising air spaces of more than 4 mm), the extent of emphysema and finally, a semiquantitative evaluation of whole scans (0 = predominant GGOs, 1 = equal distribution, 2 = predominant reticular patterns). HRTC was performed in both prone and supine positions in order to exclude the contribution of gravity to the images. We preferred to use the Wells score in order to obtain a semiquantitative evaluation of ILD-SSc, with the possibility of distinguishing the amount of fibrosis and GGOs. The value of the Wells score was evaluated in a blind experiment by two independent, experienced radiologists, reporting good concordance between them (inter-reader agreement 0.63).

The nailfold videocapillaroscopy (NVC) was performed during the visit, by two clinicians. The nailfold capillaries of all fingers of both hands, excluding thumbs, were examined in each patient using a videocapillaroscopy with a 200× magnification lens. Four consecutive 1 mm fields for a total extension of 4 mm in the middle of the nailfold were examined. The derived digital images were then stored and analysed using dedicated software (Videocap Scalar Co., Ltd., DS MediGroup, Milan, Italy). Each NVC was classified as “early”, “active” or “late” according to the method proposed by Cutolo [16]. In addition, we considered the quantitative number of synchronous microhemorrhages, microthrombosis, and giant capillaries according to the number of microhemorrhages (NEMO) score, a validated index to evaluate DA in SSc according to NVC findings [17,18,19].

General blood tests were also carried out for the complete blood count, ESR, C-reactive protein, creatinine, creatine phosphokinase, complement fractions C3 and C4, antinuclear antibodies in indirect immunofluorescence, extractable nuclear antigen antibodies with a commercial ELISA kit comprising SSA, SSB, anticentromeric antibodies (ACA), anti-topoisomerase I autoantibodies (Scl70), anti-Sm and anti-ribonucleoproteins.

To complete the clinical assessment, the patients also underwent a transthoracic echocardiogram to obtain an estimation of systolic pulmonary artery pressure (SPAP) and pulmonary function tests, including DLCO. Both these tests were performed by expert operators independent of this study. DLCO was preferred to Forced Vital Capacity for its better sensitivity and for its inclusion in the ESSG criteria [4,12,20,21].

A statistical analysis was performed by standard procedures using IBM SPSS Statistics for Windows, Version 20.0 (IBM Corp., Armonk, NY, USA). A D’Agostino–Pearson test was performed in order to evaluate their distribution. Since the values obtained did not appear to have a normal distribution, we used non-parametric tests to compare these variables with other categorical or ordinal variables taken into account in the study, (Mann–Whitney U test and Spearman’s test were used for correlation).

This study was conducted according to the Helsinki Declaration, updated to the latest version (approval by Ethical Committee Milan area 2 cod. 929_2019bis, 11 October 2019), and a written informed consent was obtained from all of the enrolled patients.

## 3. Results

We enrolled 26 dcSSc and 14 lcSSc patients. The general features of these patients are reported in Table 1.

The TE-ILD was higher in patients with high ESR (*p* = 0.03) and a referred worsening of the cardio-pulmonary function and dyspnea (ΔCP) (*p =* 0.01). Considering ILD features separately, both fibrosis and GGOs were higher in patients with ΔCP (*p =* 0.02 and 0.01, respectively). However, we did not find any correlation between the quantification of ILD involvement and the ESSG index.

When we considered the disease duration, and taking into account the early form, both TE-ILD and GGOs were higher in patients with scleredema (*p =* 0.03 and *p =* 0.01, respectively) and with ΔCP (*p =* 0.03 and 0.009). No correlation was found between the early form of SSc and DA, mRSS, SPAP or DLCO. In the longstanding form, TE-ILD, fibrosis and GGOs were higher in patients with an elevation of ESR (*p =* 0.004, 0.008 and 0.02, respectively). We also found a significant correlation between the grade of fibrosis and SPAP (r = 0.42, *p =* 0.04).

**Figure 1:**
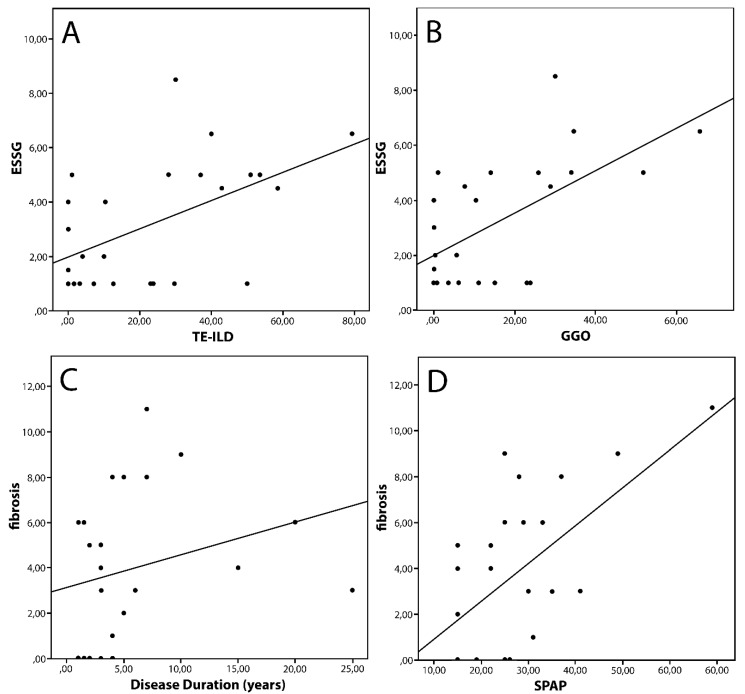
Correlation in diffuse cutaneous Systemic Sclerosis. Legend: ESSG: European Scleroderma Study Group Index; GGO: Ground Glass Opacity; SPAP: Systolic Pulmonary Artery Pressure TE-ILD: Total Extent of Interstitial Lung Disease. Spearman’s Test (**A**) r = 0.45, *p* = 0.02; (**B**) r = 0.5, *p*= 0.009; (**C**) r = 0.4 *p* = 0.04; (**D**) r = 0.46, *p* = 0.02.

Taking into account only dcSSc, we found that TE-ILD, fibrosis and GGOs were higher in patients with scleredema (*p =* 0.009, 0.05 and 0.04 respectively). In this group of patients, TE-ILD was higher in patients with ΔCP (*p* < 0.0001), while patients with an elevation of ESR had higher amounts of GGO (*p =* 0.04). We also found correlations between the ESSG index and both TE-ILD and GGO (r = 0.45 *p =* 0.02 and r = 0.5 *p =* 0.009). Fibrosis correlated with disease duration (r = 0.4 *p =* 0.04) and with SPAP (r = 0.46 *p =* 0.02) (Figure 1). Detailed results are reported in Table 2.

No significant results were found in patients with lcSSc.

NVC scores were not associated with lung involvement in both the disease subsets.

## 4. Discussion

ILD is one of the most important clinical challenges for both rheumatologists and pulmonologists in the management of SSc patients. Although the NSIP pattern is prevalent, HC areas are not uncommon, and the UIP pattern is the second most prevalent.

In our study, population ILD was associated with the worsening of respiratory symptoms (ΔCP) and higher levels of ESR. The lack of correlation between mRSS and TE-ILD could be explained by an independent progression of skin thickening and fibrosis, in accordance with what has been observed by Shand et al. [22].

In our study, the ESSG DA score was not associated with the TE-ILD in the overall cohort. This data could be explained by the presence of lcSSc patients, in which the pulmonary involvement is significantly lower than what was observed in dcSSc patients. In fact, ACA (a serological marker of lcSSC) appears to be protective for a clinically severe pulmonary involvement, whereas the anti-Scl70 antibody is associated with a faster and more severe evolution of ILD [23]. Indeed, in our cohort, DA in lcSSc patients is expressed by items that are not associated with the lung.

To confirm this, taking into account only dcSSc patients, the ΔCP and scleredema were associated with the presence of a higher amount of HC, GGO and TE-ILD, and the ESSG DA score was associated with TE-ILD and the amount of GGOs (the latter associated with high ESR), while fibrosis correlated with DD and SPAP. The association between fibrosis and DD could be explained by a progression from GGO to HC, and therefore fibrosis can be an expression of the lung disease severity (generated by the sum of the flares of the disease during the clinical course), rather than of DA. On the other hand, although SSc is obviously able to produce vasculopathy with a direct pathogenic mechanism, the association of fibrosis and a higher level of SPAP could be explained by an indirect mechanism linked to the loss of the interstitial space of the lung. This mechanism may be similar to what is expected in idiopathic pulmonary fibrosis (IPF) [24]. On the other hand, the association between the ESSG index and the proportion of GGOs can support the hypothesis that this radiological sign can represent pulmonary inflammatory DA in dcSSc. The association between DA index and TE-ILD can be due to the simple high proportion of GGOs in these patients.

The NEMO score should be mentioned, as it is of high interest. This is a validated NVC index that is able to predict, in an easy way, DA in SSc. NVC demonstrated good performance in the follow-up of SSc patients and could be useful for selecting patients for whom a more aggressive immunosuppressive treatment is appropriate. However, in our study, NEMO was not related with any parameters of lung involvement in our patients. This data can be explained by the small cohort of patients involved, but also for an intrinsic limit of the score. Probably NEMO is more sensible when recognizing vascular and cutaneous features rather than pulmonary DA. It should be taken into account that in the ESSG DA index, pulmonary DA is poorly explored: the respiratory items are DLCO <80% of the predicted (weight 0.5 out of 10) and ΔCP (weight 2 out of 10). Both these conditions can be secondary to pulmonary artery hypertension, rather than ILD, reflecting a vascular involvement.

A fascinating topic is also the quantification of lung involvement in SSc. An objective evaluation of ILD can be useful for both clinical and research purposes. Actually, the majority of clinical trials on ILD-SSc use pulmonary function tests (PFTs) as a surrogate primary outcome, but the performance of these parameters is often burdened by a concomitant condition in SSc (e.g., PAH) [4]. A number of methods were reported in the quantification of ILD-SSc, generally divided into semi-quantitative and quantitative [25]. Semiquantitative methods have the merit to discriminate GGOs (possible active alveolitis) by HC (expression of fibrotic damage) and are relatively easy to perform in clinical practice, but they are limited by operator-dependence and they may not be sufficiently sensitive for minimal changes in lung damage. Quantitative methods overcome the inter-observer grade of concordance, but are not able to distinguish the main feature of lung damage. Moreover, both semiquantitative and quantitative methods were associated with mortality and PFTs at baseline [25,26,27], but this correlation was not always confirmed in the follow-up. A possible explanation is that a reduction in the total amount of GGOs, with a consequent increase in the proportion of HC, could not change quantitative values, but it is associated with an impairment of PFTs.

The aim of our work was to evaluate the clinical significance of the two main features in ILD-SSc (GGOs and HC) and therefore we preferred a semi-quantitative score. However, interesting methods were recently reported by Bocchino M et al. [28]. The authors developed a composite index, taking into account mean lung attenuation, skewness and kurtosis, in low dose volumetric HRCT. This index was associated with inflammatory and PFT parameters at baseline and at one-year follow-up. Moreover, this score was also able to recognize minimal changes in both the cohort of SSc studied, with and without ILD. The authors hypothesized that this score could be useful to predict ILD in SSc without lung involvement at baseline. The texture analysis resulted in being useful to combine the advantage of quantitative assessment, with the possibility of distinguishing GGOs from HC [29]. Unfortunately, this method requires very sophisticated tools that are not widely available in clinical practice, and need a prospective validation.

However, HRCT quantification seems to be a promising tool, and it is reasonable to imagine that future clinical trials in ILD-SSc will consider it as a possible primary outcome.

Finally, the study has some limits due to the limited number of patients enrolled, and the good but not absolute inter-reader agreement in the semi-quantitative evaluation of ILD-SSc. However, the extensive clinical and instrumental assessment, as well as the correlation with DA, could highlight the value of a different approach in the management of ILD-SSc.

Our study shows that in dcSSc patients, high disease activity scores are associated with a high proportion of ILD and GGOs, but not fibrosis. Possible predictors of lung involvement in these patients are the presence of scleredema, high ESR and ΔCP. These data can be useful, not only in recognizing active lung disease in SSc patients, but also in selecting patients for whom an aggressive immunosuppressive treatment can be useful. In this view, the lack of association between disease activity and fibrosis can be useful. In reality, at the moment, the treatment of ILD-SSc has produced conflicting results, despite the large number of compounds studied [30]. The lack of significant results in clinical trials can be explained by the absence of validated primary outcomes (e.g., PFTs parameters [31]), but a role could be played by the frequent absence of stratification according to the HRCT pattern. A prevalent GGO involvement could be an expression of DA and it might benefit from aggressive immunosuppression, whereas in the presence of HC areas or even a UIP pattern, this treatment could be detrimental, as reported for idiopathic pulmonary fibrosis [32]. An aggressive immunosuppression was not able to revert fibrotic lung damage in IPF or SSc however, remaining associated with the known side effects [33]. These patients can benefit from the use of conventional therapy (controlling systemic DA) associated with anti-fibrotic drugs, as recently reported for Nintedanib in SSc [34].

## Figures and Tables

**Table 1:** Demographic data and clinical and radiographic features of the 40 patients with Systemic sclerosis, according to the European Scleroderma Study Group (ESSG) score and Wells score.

	Whole Cohort	Patients with Diffused Form Systemic Sclerosis (dcSSc)	Patients with Limited Form Systemic Sclerosis (lcSSC)	*p*
**Total patients (gender F/M)**	40 (36/4)	26 (23/3)	14 (13/1)	n.s.
**Age (years)**	54 (47–65)	51.5 (46–67.5)	56.5 (51–66.5)	n.s.
**Disease duration (years)**	5 (2.75–7)	4 (3–7)	6 (3–9.75)	n.s.
**mRSS**	4 (1–8)	4 (1.75–8)	3 (0.5–6)	n.s.
**Scleredema (%)**	65	65.3	64.2	n.s.
**Δ Skin (%)**	30	30.7	28.4	n.s.
**Ulcers (%)**	22.5	26.9	14.2	n.s.
**Δ Vascular (%)**	25	19.2	35.7	n.s.
**Arthritis (%)**	7.5	11.5	0	<0.001
**DLCO <80 of the predicted (%)**	85	88.4	78.5	n.s.
**Δ cardiopulmonary (%)**	32.5	34.6	28.6	n.s.
**ESR >30 (%)**	40	53.8	14.3	0.01
**Hypocomplementemia (%)**	7.5	3.8	14.3	n.s.
**ESSG index**	3 (1–4.5)	3.5 (1–5)	3.5 (0.5–4.5)	n.s.
**SPAP**	25 (15–32)	25 (20–31)	20 (15–33.5)	n.s.
**ILD (overall extent)**	7.3 (1.1–28)	23.5 (1.6–41.5)	1.2 (0–2.35)	0.0008
**Quantification of GGOs**	5.5 (0–23)	10.65 (1–27.3)	0.3 (0–1.6)	0.003
**Grade of fibrosis**	3 (0–5)	4 (0–7)	0.5 (0–3)	0.007

If not specified, all data are considered in median (minimum, first, third quartiles, maximum). Legends: Δ: variation (worsening); DLCO: diffusion lung Carbone monoxide; ESR: erythrocyte sedimentation rate; ESSG index: European Scleroderma Study Group Index; SPAP: systolic pulmonary artery pressure; ILD: interstitial lung disease; GGOs: ground glass opacities; mRSS: modified Rodnan skin score, n.s.: not significant. Data are reported in median, Inter Quartile Range, (IQR).

**Table 2:** Significant differences in semi-quantitative assessment of ILD according to the items included in ESSG score.

Whole Cohort of Patients			
**Total Extent ILD**	**No**	**Yes**	***p***
**ΔCP median (** ***n*** **)**	3.6 (27) (0.5–12.6)	37 (13) (1–51)	0.01
**ESR median (** ***n*** **)**	16.7 (21) (0–16.7)	29 (19) (1.8–29)	0.03
**GGO**	**No**	**Yes**	***p***
**ΔCP median (** ***n*** **)**	1.2 (27) (0–8.25)	25.8 (13) (2–34)	0.02
**Fibrosis grade**	**No**	**Yes**	***p***
**ΔCP median (** ***n*** **)**	2 (27) (1–4)	5 (13) (2–8)	0.01
**Early Systemic Sclerosis Total Extent ILD**	**No**	**Yes**	***p***
**Scleredema median (** ***n*** **)**	0 (7) (0–3)	13.9 (11) (1.4–37.5)	0.03
**ΔCP median (** ***n*** **)**	1.2 (14) (0–4)	35 (4) (30–40)	0.03
**GGO**	**No**	**Yes**	***p***
**Scleredema median (** ***n*** **)**	0 (7) (0–1.5)	13 (11) (1.4–28.7)	0.01
**ΔCP median (** ***n*** **)**	1 (14) (0–6.2)	32.3 (4) (30–34)	0.009
**Longstanding Systemic Sclerosis Total Extent ILD**	**No**	**Yes**	***p***
**ESR > 30 median (** ***n*** **)**	5 (14) (0.2–15.7)	37 (9) (10–51)	0.004
**GGO**	**No**	**Yes**	***p***
**ESR > 30 median (** ***n*** **)**	2 (14) (0–3.75)	8 (9) (3–9)	0.008
**Fibrosis**	**No**	**Yes**	***p***
**ESR > 30 median (** ***n*** **)**	2.14 (14) (0–9.2)	14 (9) (5.1–28.8)	0.02
**Diffuse Cutaneous Systemic Sclerosis Total Extent ILD**	**No**	**Yes**	***p***
**Scleredema median (** ***n*** **)**	1.6 (9) (0–7.2)	30 (17) (10.3–50)	0.009
**ΔCP median (** ***n*** **)**	4 (17) (0–12.6)	42.9 (9) (37–53.5)	<0.0001
**GGO**	**No**	**Yes**	***p***
**Scleredema median (** ***n*** **)**	0.68 (9) (0–6)	15 (17) (7.6–30)	0.007
**ESR > 30 median (** ***n*** **)**	6.2 (11) (2.14–13)	14 (15) (0.6–32)	0.04
**Fibrosis**	**No**	**Yes**	***p***
**Scleredema median (** ***n*** **)**	2 (9) (0–3)	5 (17) (3–8)	0.05

ΔCP: variation cardiopulmonary symptoms (worsening); ESR: erythrocyte sedimentation rate; ESSG index: European Scleroderma Study Group Index; ILD: interstitial lung disease; GGOs: ground glass opacities. The features not reported in table resulted in being not statistically significant.

## Abbreviations

ACR/EULAR	American College of Rheumatology/European League Against Rheumatism
Scl70	Anti-topoisomerase I autoantibodies
ACA	Anti-centromeric antibodies
dcSSc	Diffuse cutaneous systemic sclerosis
DLCO	Diffusion lung for carbon monoxide
DA	Disease activity
DD	Disease duration
ΔCP	Dyspnea
ESR	Erythrocyte sedimentation rate
GGOs	Ground-glass opacities
HRCT	High resolution computed tomography
HC	Honeycombing
ILD	Interstitial lung disease
lcSSc	Limited cutaneous systemic sclerosis
mRSS	Modified Rodnan skin score
NVC	Nailfold videocapillaroscopy
NEMO score	Number of microhemorrhages score
NSIP	Nonspecific interstitial pneumonia
RP	Raynaud’s phenomenon
SSc	Systemic sclerosis
SPAP	Systolic pulmonary artery pressure
TE-ILD	Total extent of ILD
UIP	Usual interstitial pneumonia
